# Comparative Pharmacokinetic Study for Linezolid and Two Novel Antibacterial Oxazolidinone Derivatives in Rabbits: Can Differences in the Pharmacokinetic Properties Explain the Discrepancies between Their In Vivo and In Vitro Antibacterial Activities?

**DOI:** 10.3390/pharmaceutics9030034

**Published:** 2017-09-07

**Authors:** Mohsen A. Hedaya, Vidhya Thomas, Mohamed E. Abdel-Hamid, Elijah O. Kehinde, Oludotun A. Phillips

**Affiliations:** 1 Department of Pharmaceutics, Faculty of Pharmacy, Kuwait University, P.O. Box 24923, Safat 13110, Kuwait; vidhyaelizabeth@gmail.com; 2 Department of Pharmaceutical Chemistry, Faculty of Pharmacy, Kuwait University, P.O. Box 24923, Safat 13110, Kuwait; abdel-hamid@hsc.edu.kw (M.E.A.-H.); dphillips@hsc.edu.kw (O.A.P.)

**Keywords:** linezolid, oxazolidinone antibiotics, pharmacokinetics, renal excretion, bioavailability, tissue distribution, microemulsion

## Abstract

This is a comparative pharmacokinetics study of linezolid (Lzd), and two novel oxazolidinone antibacterial agents—PH027 and PH051—in rabbits to determine if the discrepancy between the in vitro and in vivo activities of the novel compounds is due to pharmacokinetic factors. The pharmacokinetics after IV and oral administration, plasma protein binding and tissue distribution for the three compounds were compared. The elimination half-lives were 52.4 ± 6.3, 68.7 ± 12.1 and 175 ± 46.1 min for Lzd, PH027 and PH051, respectively. The oral bioavailability for Lzd, PH027 and PH051 administered as suspension were 38.7%, 22.1% and 4.73%, which increased significantly when administered as microemulsion to 51.7%, 72.9% and 13.9%. The plasma protein binding were 32–34%, 37–38% and 90–91% for Lzd, PH027 and PH051. The tissue distribution for PH027 and PH051 in all investigated tissues were higher than that for Lzd. It can be concluded that the lower bioavailability of PH027 and PH051 compared to Lzd when administered as suspension is the main cause of their lower in vivo activity, despite their comparable in vitro activity. Differences in the other pharmacokinetic characteristics cannot explain the lower in vivo activity. The in vivo activity of the novel compounds should be re-evaluated using formulations with good oral bioavailability.

## 1. Introduction

The increasing incidence of bacterial resistance created an urgent need for the discovery and development of new classes of antibiotics that are more effective and safer than currently existing agents. Despite the massive research efforts in this area, very limited number of novel antibacterial classes have been marketed over the past 30 years. The oxazolidinones are a new class of antibacterial drugs, initially discovered in the 1980s, leading to the approval of the first member of this class, Linezolid (Lzd), Zyvox^®^. Lzd was approved in 2000 by the US Food and Drug Administration (FDA) for the treatment of infections caused by Gram-positive resistant bacteria including vancomycin-resistant enterococci, Methicillin-resistant *Staphylococcus aureus*, nosocomial pneumonia, community-acquired pneumonia, and complicated and uncomplicated skin infections [1]. Oxazolidinones are attractive class of compounds because they have relatively low frequency of resistance [2]. Despite the extensive research efforts to develop safer and more effective derivatives, linezolid remained the only approved drug in this class of compounds for about 15 years [3,4,5].

Several oxazolidinone derivatives have been investigated, but their clinical trials were terminated because of problems related to insufficient efficacy, unfavorable pharmacokinetic or physicochemical properties, poor safety profile, and/or lack of superiority over Lzd. Currently there are several oxazolidinone derivatives at various stages of clinical trials to evaluate their safety and efficacy profiles [5]. Ideally, newly-prepared oxazolidinones should have improved efficacy, wider spectrum of activity, shorter course of therapy, improved safety profile and the possibility for use by different routes of administration such as intravenous (IV) and oral dosing. A second member of this class, tedizolid phosphate, which can be considered a second-generation oxazolidinone has been approved by the US FDA recently. This drug is effective against linezolid-resistant bacterial strains and its pharmacokinetic properties allow once daily dosing [6,7].

Our group have synthesized several novel triazolyl-oxazolidinone derivatives and evaluated their antibacterial activity against clinical isolates of bacterial strains. Some of these compounds showed good in vitro antibacterial activity against these bacterial strains with minimum inhibitory concentrations (MIC), which was comparable and/or superior to those of Lzd [8,9,10,11,12]. Examples of the newly prepared triazolyl oxazolidinone compounds include; PH027, a morpholine derivative, and PH051, a piperazine derivative, that have shown good in vitro antibacterial activity. For *Staphylococcus aureus* and Enterococci spp, the MIC for Lzd, PH027 and PH051 were 0.5–1.0, 0.5–1.0 and 0.25–0.5 mg/L, respectively. While for *Escherichia coli,* the MIC for the three compounds were >64 mg/L, and for *Heamophilus influenzae* the MIC were 832 and 64 for Lzd, PH027 and PH051, respectively, the MIC for Lzd, PH027 and PH051 for *Streptococcus pneumoniae* were 0.5 mg/L, 0.5 mg/L and 1.0 mg/L, and for *Moraxella catarrhalis* were 8.0, >16.0 and 8.0 mg/L, respectively. However, investigation of the in vivo efficacy of selected triazolyl-oxazolidinone derivatives, in systemic infection model showed conflicting results. The mice were infected with *S. aureus* Giorgio, (CFU/mouse 5 × 10^7^) which were sufficient to kill 100% of the infected mice in 24 h. The compounds were administered orally in the form of suspension to the mice 1 and 4 h after induction of the infection, and the survival of mice was recorded for 72 h. The in vivo activity of Lzd against systemic mice infection was better than the in vivo activity of the novel triazolyl-oxazolidinone derivatives, PH027 and PH051, following oral administration despite their comparable in vitro antibacterial activities (unpublished data from Jin-Hwan Kwak at Handong Global University, Pohang Gyeongbuk, Korea).

Generally, the pharmacokinetic properties of drugs such as absorption, distribution, and elimination are important in determining the in vivo efficacy of systemically acting drugs including antibiotics [5,13]. This is because the drug must be absorbed into the systemic circulation in sufficient quantity after extravascular administration to produce its effect, otherwise the drug has to be administered intravenously. Additionally, the drug distribution characteristics, which depends on the affinity of the drug to the different tissues, are important in determining the drug concentration at the target organ. Furthermore, the rate of drug elimination which affects the rate of decline in the drug concentration in the various parts of the body, is important in determining how long the drug concentration will stay above the desired concentration. In general, the goal of successful antibiotic therapy is to maintain the drug concentration at the infected site(s) above the drug MIC for the pathogen of interest, for an adequate duration of time. The pharmacokinetic behavior of the antibiotic is crucial in achieving this goal.

This comparative pharmacokinetic study was designed to investigate whether the lower in vivo antibacterial activity of the two novel derivatives, as compared to Lzd, despite their comparable in vitro activity, is resulting from pharmacokinetic factors. The main objective was to compare the pharmacokinetic characteristics of Lzd, PH027 and PH051 using the rabbit as the animal model. This includes the general pharmacokinetic behavior after IV administration and oral administration as suspension and microemulsion, the main route of elimination, the blood-to-plasma partition, the plasma protein binding, and the tissue-to-plasma distribution ratio of the three compounds. The general pharmacokinetic characteristics of the three compounds were compared to determine if the difference in any of the pharmacokinetic parameters can contribute to the lower in vivo activity of the novel compounds.

## 2. Materials and Methods

### 2.1. Chemicals

All chemicals used for synthesis of Lzd, PH027, and PH051, and for the sample analysis were purchased from Sigma-Aldrich (Darmstadt, Germany). Cremophor-EL, Solutol HS, and Captex 355, were obtained from ABITEC Corporation (Janesville, WI, USA). MilliQ water was used throughout the whole work. All chemicals were of commercial grade and the solvents (acetonitrile, methanol) were of High Performance Liquid Chromatographic (HPLC) grade.

### 2.2. Synthesis of Lzd, PH027, and PH051

**Figure 1:**
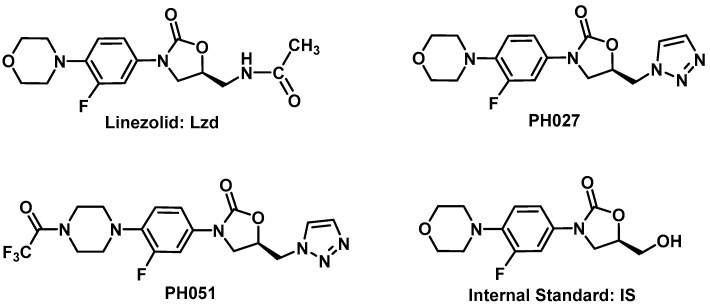
Chemical structure for linezolid (Lzd), PH027, PH051 and the Internal Standard (IS) used in the analysis.

Lzd, PH027, PH051, and the Internal Standard [IS, (*R*)-3-(3-fluoro-4-morpholinophenyl)-5-(hydroxymethyl) oxazolidin-2-one], used in the assay, shown in Figure 1, were synthetized in our laboratory utilizing the synthesis schemes reported previously [8,9,14]. All the synthetized compounds were characterized by ^1^H-NMR, ^13^C-NMR, MS, IR, and CHN analysis.

### 2.3. Experimental Animals

White New Zealand rabbits, about 10 weeks old, weighing 3.0–3.5 kg were used in these studies. Rabbits were obtained from the Animal Resource Center at the Health Science Center, Kuwait University. They were kept individually in stainless steel cages with free access to food and water, in temperature controlled room under 12 h light/dark cycles. The protocols for the animal experiments were approved by the Animal Care and Use Committee at the Health Science Center, Kuwait University. The rabbit was chosen as the animal model because it allows serial urine and plasma sampling after IV and oral administration, and this model has been used extensively in pharmacokinetic investigations, including bioavailability studies.

#### 2.3.1. Intravenous Administration and Collection of Blood Samples

IV administration of the compounds under investigation, and collection of blood samples were through a catheter inserted in the marginal ear vein of the rabbit (I-Cath, CharterMed Inc., Winston-Salem, NC, USA) [15,16]. After IV drug administration, blood was withdrawn to fill the cannula and then reinjected three times before obtaining any blood samples to prevent contamination. Furthermore, the cannula was flushed with heparinized saline to prevent blockage of the cannula and prevent sample to sample contamination.

#### 2.3.2. Urine Collection

Urine samples were collected utilizing Foly Catheter (size French 8). The catheter was inserted into the rabbit bladder using a lubricant. When urine is collected over a specific time interval, the bladder was irrigated with 3 × 15 mL normal saline at 3 min, 6 min, and 10 min before the end of the urine collection interval [15,16]. Irrigation of the bladder is necessary to obtain the majority of the drug eliminated in urine during the collection interval. This usually causes dilution of the urine samples but does not affect the results since the amount excreted during the collection interval is calculated from the product of the urine drug concentration and sample volume.

#### 2.3.3. Oral Administration

Oral administration of the compounds under investigation was through a pediatric feeding tube after opening the rabbit mouth with a rubber tube that has two holes facing each other. The pediatric feeding tube was pushed until it reached the stomach.

#### 2.3.4. Euthanasia

After the end of each experiment, the rabbits were euthanized using an overdose of IV pentobarbital [15,16].

### 2.4. Blood to Plasma Partition

Aliquots of 2 mL of freshly drawn heparinized rabbit blood were spiked with known amounts of each of the three compounds to produce final whole blood concentrations of 250 μg/L, and 1000 μg/L (*n* = 3 for each concentration for each compound). The spiked blood samples were kept at 37 °C in a shaking water bath for one hour, and plasma was then obtained by centrifugation at 37 °C. Aliquots of about 400 μL, of each plasma sample were kept frozen at −80 °C until analysis. The blood to plasma distribution ratio was determined from the ratio of the nominal total whole blood concentration and the measured plasma concentration for each compound.

### 2.5. Plasma Protein Binding

The remaining volume of the plasma samples obtained by centrifugation in the blood to plasma partition experiment, about 600 μL, was immediately placed in disposable ultrafiltration devices (Centrifree^®^, EMD Millipore Corp., Billerica, MA, USA), and centrifuged in a temperature controlled centrifuge at 37 °C. The ultrafiltrate collected in the ultrafiltration device cup was stored at −20 °C until analysis. The plasma protein binding for the three compounds in rabbit plasma was determined in triplicates at two different concentrations. The total (free + bound) plasma concentration is the concentration in the plasma samples obtained by centrifugation in the blood to plasma partition experiment, while the free (unbound) concentration is the concentration in the ultrafiltrate. The percentage bound was calculated from the free concentration and the corresponding total concentration according to Equation (1).

(1) $$\%\;\mathrm{Bound}\;=\;\frac{\mathrm{Total}\;\mathrm{concentration}-\mathrm{Free}\;\mathrm{concentration}}{\mathrm{Total}\;\mathrm{concentration}}\;\times \;100$$

### 2.6. Pharmacokinetics after Single IV Administration

Three groups of rabbits (*n* = 4, each) received 5 mg/kg dose of each compound via the IV catheter. This dose was selected based on the results of a preliminary experiment to achieve measurable plasma concentrations for 8 h using our analytical methods, to ensure good characterization of the plasma concentration-time profile. Accurately weighed Lzd and PH027 powder were dissolved first in 600 μL of ethanol using an ultrasound water bath. The resulting solution was then diluted with normal saline to produce the solutions used for IV administration, which consist of the dose of the compound dissolved in 3 mL of 20% ethanol in normal saline. PH051 required the use of slightly larger volume of ethanol to achieve complete dissolution. So, 900 μL of ethanol were used, and the resulting solution used for IV administration consisted of PH051 dose dissolved in 3 mL of 30% ethanol in normal saline. The difference in the volume of ethanol administered, 300 μL, which was necessary to ensure complete dissolution of PH051, should not have a significant effect on the pharmacokinetic behavior of PH051. The solutions were administered slowly over a period of 4–5 after administration, serial blood and urine samples were obtained over a period of 8 h. Plasma was obtained from blood by centrifugation, and samples were kept at −80 °C until analysis. The volumes of urine samples were determined and recorded, and aliquots were kept at −80 °C until analysis.

### 2.7. Pharmacokinetics after Single Oral Administration

Three groups of rabbits (*n* = 4, each) received 20 mg/kg oral dose of each compound. This dose was higher than the IV dose, because the results of our preliminary experiments showed that the three compounds have incomplete absorption, and we wanted to ensure good characterization of the plasma concentration-time profile after oral administration. Accurately weighted amounts of each compound were dissolved/suspended in 20 mL of warm normal saline using an ultrasound water bath. The three compounds did not dissolve completely in the solution used for oral administration, as these solutions were not clear when they were left at room temperature before administration. However, it was clear that Lzd dissolved better than PH027, and PH051 had the lowest solubility among the three compounds in normal saline. The solutions/suspensions of the three compounds were administered orally to the rabbits using a pediatric feeding tubes. After oral administration of each compound, serial blood samples were obtained through the IV catheter, over a period of 10 h after administration. Plasma was obtained from blood by centrifugation, and samples were kept at −80 °C until analysis.

### 2.8. Pharmacokinetics after a Single Oral Administration in the Form of Microemulsion

Because the oral dose of three compounds did not completely dissolve in normal saline before oral administration, an attempt was made to administer the compounds under investigation in the form of microemulsion, using self-emulsifying drug delivery systems (SMEDDS). The compounds were dissolved in 3 mL of Cremophor-EL, Solutol HS, Captex 355 (6:3:1), then this solution was diluted with 12 mL of distilled water just before drug administration, as described previously for other lipophilic compounds [17]. Three groups of rabbits (*n* = 3, each) received 20 mg/kg oral dose of each compound in the form of microemulsion through a pediatric feeding tube. After administration, serial blood samples were obtained through the IV catheter, over a period of 10 h. Plasma was obtained from blood by centrifugation, and samples were kept at −80 °C until analysis.

### 2.9. Tissue to Plasma Distribution

Three groups of White New Zealand rabbits (*n* = 3, each) weighing 3.0–3.5 kg were used. Each group received 10 mg/kg of one compounds administered intravenously over a period of 3–5 min, followed one hour later by a second IV dose of 10 mg/kg. Two h later, the rabbits were euthanized with an IV dose of pentobarbital. Plasma and tissue samples, including kidney, liver, lungs, spleen, heart, muscles, and vetrus fluid were collected immediately, and stored at −80 °C until analysis.

### 2.10. Plasma and Urine Sample Analysis

Analysis of urine, plasma and tissue homogenate samples was performed using Waters Acquity UPLC H-Class-Xevo TQD system (Milford, MA, USA), equipped with an electrospray ionization probe operated in the positive ionization mode. Chromatographic separation of analytes was carried out on Acquity UPLC BEH C18 (50 mm × 2.1 mm, 1.7 μm) column using a mobile phase of 2 mM ammonium acetate buffer solution and acetonitrile (70:30) at a flow rate of 0.3 mL/min for Lzd and PH027. For PH051, the mobile phase was 0.25% formic acid in water, and acetonitrile (10:90), at a flow rate of 0.2 mL/min. The source dependent parameters maintained for the analytes and internal standard were: cone gas flow, 50 L/h; desolvation gas flow, 800 L/h. The optimum values for compound dependent parameters like source desolvation temperature, capillary source voltage, cone voltage and collision energy were set at 500 °C, 3.50 kV, 35 V and 27 V for Lzd; 300 °C, 3.26 kV, 36 V and 25 V for PH027; 500 °C, 3.95 kV, 46 V and 66 V for PH051; and 300 °C, 3.5 kV, 30 V and 25 V for the IS, respectively.

Unit mass resolution was employed and the dwell time was set at 100 ms for Lzd, PH051, and IS, and at 25 ms for PH027. Quantitative analysis was performed using multiple-reaction monitoring (MRM) scanning mode, by determining the transition ranges (precursor > product mass ions) of *m*/*z* 338.30 > 296.08 for Lzd, *m*/*z* 348.24 > 178.23 for PH027, *m*/*z* 443.27 > 148.27 for PH051 and *m*/*z* 297.17 > 163.17 for the internal standard. MassLynx software version 4.1 was used to control all parameters of UPLC and MS/MS.

An aliquot of 200 μL of rabbit’s plasma or urine samples was mixed with 100 μL of methanol, and 30 μL of internal standard solution (Figure 1) in methanol (10 μg/L). The mixture was extracted with 600 μL of diethyl ether for 5 min, and then centrifuged for 10 min at 13200 rpm. The ether layer was transferred to clean Eppendorf tubes, evaporated under nitrogen at room temperature in a sample concentrator. The residue was reconstituted in 300 μL of the mobile phase and the resulting solution was filtered through a 0.22 μ syringe filter and 10 μL aliquot was injected into the UPLC-MS/MS. Calibrators were prepared by spiking 200 μL of blank rabbit’s plasma or urine with 100 μL of the working methanolic solutions of the compounds under investigation to produce standard solutions in the concentration range of 50–5000 μg/L. The standard samples were then proceeded as described for sample analyses.

Plasma ultra-filtrate samples were analyzed using the same procedures for the plasma. Tissue homogenates were prepared by adding 1 gm of the tissues samples to 4 g of phosphate buffer, pH 7.4 (1:4, *w*/*w*), then homogenization using Tissue-Tearor, Bio-Spec Products Inc. (Bartlesville, OK, USA). Aliquots of 200 μL of tissue homogenate samples were analyzed using the procedures for the plasma and the concentrations were determined using plasma calibrators as described and validated previously for Lzd and PH027 [18]. 

### 2.11. Pharmacokinetic and Statistical Analysis

The pharmacokinetic parameters for each of the three compounds including half-life (t_1/2_), volume of distribution (Vd), elimination rate constant (k), area under the plasma concentration-time curve (AUC), total body clearance (CL_T_), renal clearance (CL_R_), and oral bioavailability (F) were determined using the general pharmacokinetic parameter calculation procedures using PKPD tools for Microsoft Excel [19]. The obtained pharmacokinetic parameters for each compound were compared using Statistical Package of Social Sciences (SPSS) to determine the difference in the pharmacokinetic behavior for the three compounds.

## 3. Results

### 3.1. Plasma and Urine Sample Analysis

**Figure 2:**
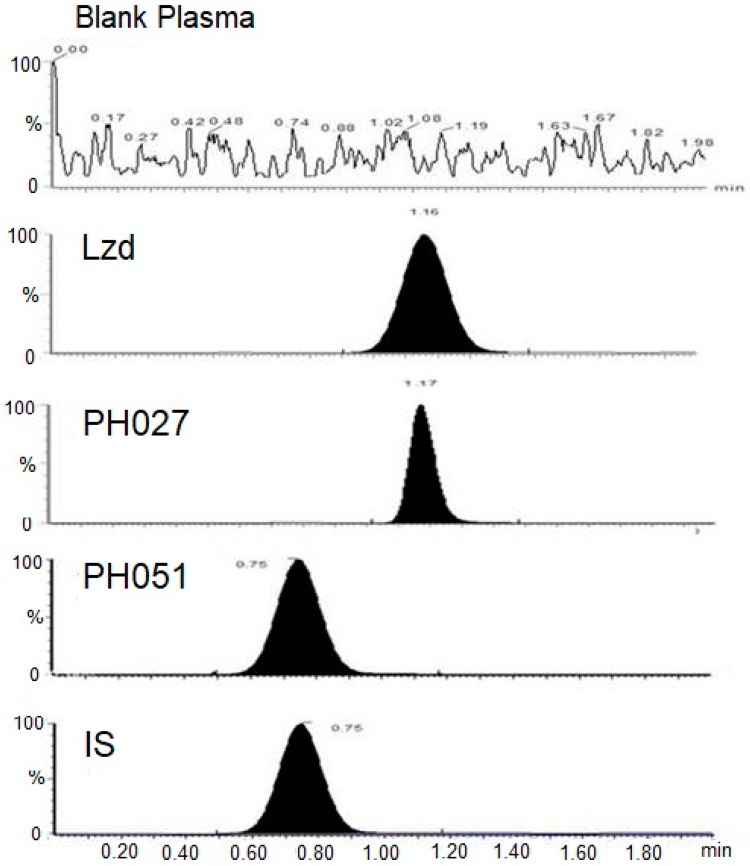
Representative chromatograms obtained from the analysis of blank plasma, Lzd, PH027 and PH051 500 μg/L standards, and the IS.

The developed UPLC-MS/MS methods for the analysis of the three compounds under investigation were proven to be accurate, precise and sensitive enough to determine the concentrations of these compounds in the biological samples obtained from the pharmacokinetic studies. The intra-day precision ranged from 1.35–5.50%, 0.76–3.50% and 1.39–6.12%, while the inter-day precision ranged from 1.84–7.60%, 5.70–13.20% and 4.33–13.28% for Lzd, PH027, and PH051, respectively. The intra-day accuracy ranged from 88.9–113.6%, 94.9–110.3% and 90.7–113.6%, while the inter-day accuracy ranged from 96.2–106.1%, 96.2–103.9% and 96.2–103.9% for Lzd, PH027, and PH051, respectively. Figure 2 represents MRM-chromatograms obtained during the analysis of blank plasma, Lzd, PH027, PH051 500 μg/L standards and the IS.

### 3.2. Blood to Plasma Partition and Plasma Protein Binding

In rabbit’s blood, the blood to plasma distribution ratio for Lzd and PH027 were not different from unity indicating equal distribution of these two compounds between blood cells and plasma. While the blood to plasma distribution ratio of PH051 was significantly lower than unity indicating higher plasma concentration compared to the total blood concentration. The plasma protein binding of Lzd and PH027 in rabbit plasma was 32–34%, and 37–38%, respectively. While the plasma protein binding of PH051 was 91%. Table 1, summerizes the blood:plasma partition and plasma protein binding of the three compunds in rabbit blood.

### 3.3. Pharmacokinetic after Single IV Administration

**Figure 3:**
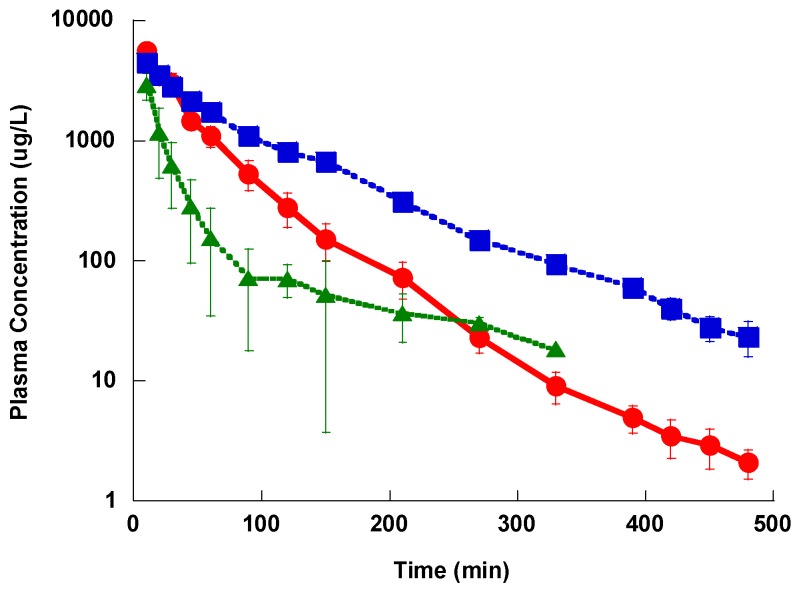
The plasma concentration-time profile for Lzd (●), PH027 (■) and PH051 (▲) after a single intravenous administration of 5 mg/kg.

**Figure 4:**
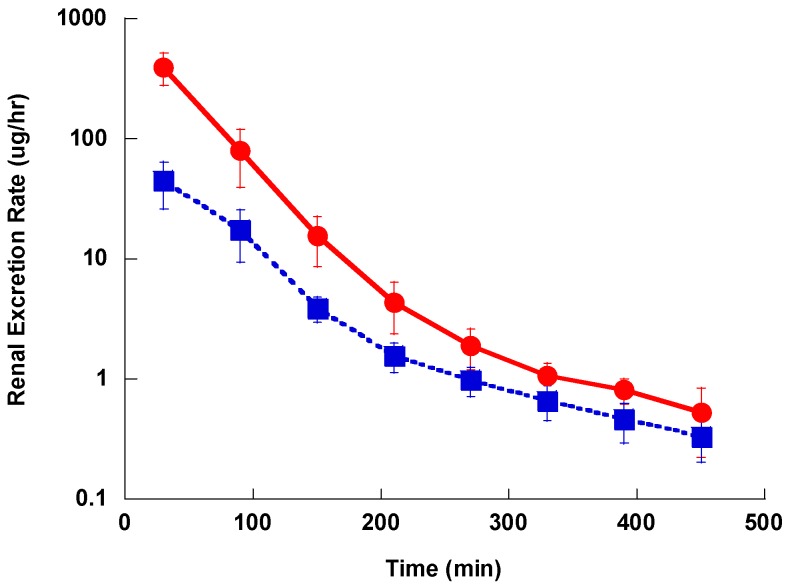
The renal excretion rate-time profile for Lzd (●) and PH027 (■) after a single intravenous administration of 5 mg/kg.

The plasma concentration-time profile after IV administration showed a distribution phase, followed by a slower terminal elimination phase with t_1/2_ of 52.4 ± 6.3, 68.7 ± 12.1 and 175 ± 46.1 min for Lzd, PH027 and PH051, respectively as shown in Figure 3. The average AUC for Lzd, PH027 and PH051 after 5 mg/kg IV administration were 4038 ± 1280, 5670 ± 933 and 1950 ± 986 μg h/L, and the calculated CL_T_ for the three compounds were 1.24 ± 0.43, 0.891 ± 0.23, and 2.564 ± 1.212 L/h/Kg, respectively. The renal excretion rate-time profile for Lzd and PH027 showed exponential decline with the rate of PH027 elimination slower than that of Lzd, as shown in Figure 4. Very little PH051 was detected in the first one or two urine collections after the IV dose. The fraction of the IV dose excreted unchanged in urine calculated from the ratio of the renal clearance to the total body clearance, were 5.70% and 0.402% for Lzd and PH027, respectively.

### 3.4. Pharmacokinetics after Single Oral Administration as Suspension and as Microemulsion

**Figure 5:**
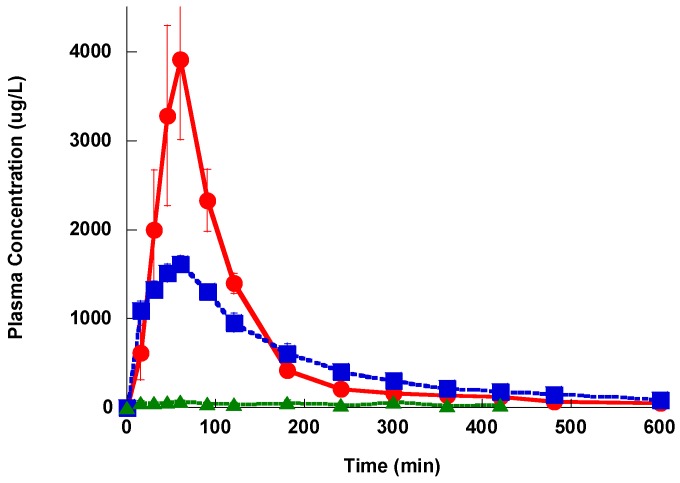
The plasma concentration-time profile for Lzd (●), PH027 (■) and PH051 (▲) after a single oral administration of 20 mg/kg.

**Figure 6:**
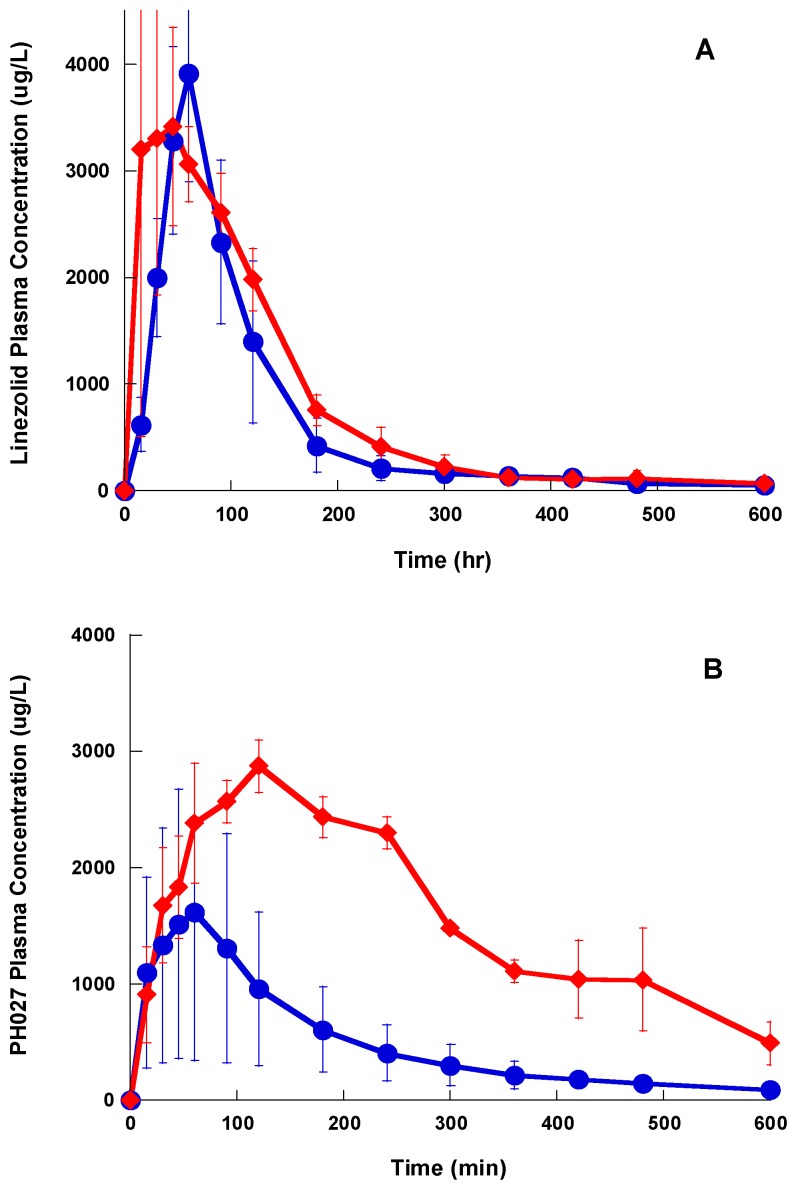

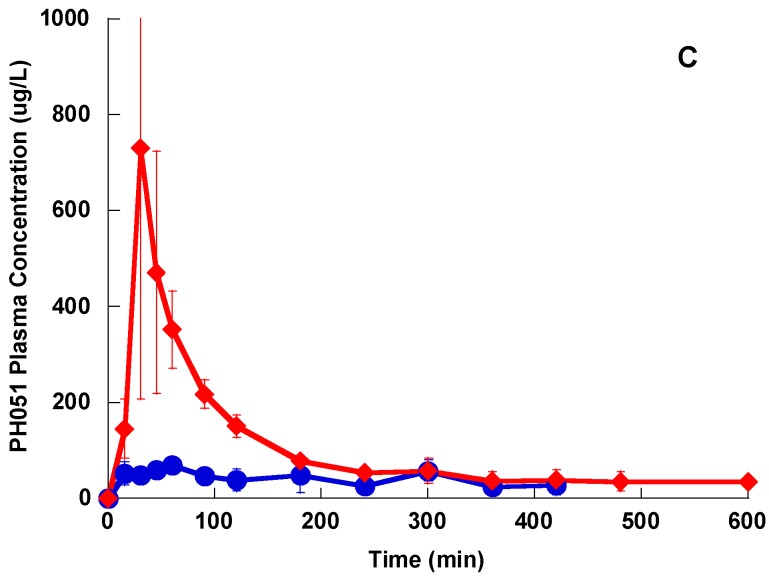
The plasma concentration-time profile for Lzd (**A**), PH027 (**B**), and PH051 (**C**), after single oral administration of 20 mg/kg in the form of suspension (●), and microemulsion (♦).

After oral administration, the maximum plasma concentrations for Lzd and PH027 were achieved in 60 min, and then the drug concentration declined exponentially, while the plasma concentrations for PH051 after oral administration were very low, as shown in Figure 5. The average AUC for Lzd after oral administration of 20 mg/kg as suspension was 6250 ± 3620 μg h/L, which indicates that Lzd oral bioavailability is 38.7%. While the average AUC for PH027 and PH051 after oral administration of 20 mg/kg as suspension were 5010 ± 2430 and 369 ± 378 μg h/L, corresponding to oral bioavailability of 22.1%, and 4.73%, respectively. Administration of the compounds in the form of microemulsion resulted in rapid absorption of PH051 and Lzd, and slower absorption for PH027, with significance increase in the AUC as shown in Figure 6A–C. The average AUC were 8350 ± 3340, 16,550 ± 3460 and 1083 ± 426 μg-h/L, for Lzd, PH027, and PH051, which correspond to oral bioavailability of 51.7%, 72.9% and 13.9%, respectively. The pharmacokinetic parameters for Lzd, PH027, and PH051in the rabbit after IV and oral administration are summerized in Table 2.

### 3.5. Tissue to Plasma Distribution

The tissue to plasma concentration ratios for the three compounds were calculated from the ratio of the tissue homogenate concentrations corrected for the dilution during the homoginization process and the plasma concentrations. Generally, the three compounds have very good distribution to all tissues. The tissue to plasma distribution ratios for the novel compounds were more than one in kidney, lung, liver, heart, muscles and spleen, and the ratios were more than those for Lzd in all investigated tissues. Table 3, summerizes the tissue to plasma distribution ratios for the three compounds in different tissues.

## 4. Discussion

Lzd is the first oxazolidinone antibiotic approved for the treatment of infections caused by Gram-positive resistant pathogenic bacteria. Since its approval, the search is continuing for newer members of this class with improved efficacy, wider spectrum activity, better safety profile, less tendency for developing resistance, and favorable pharmacokinetic characteristics. The two compounds PH027 and PH051 investigated in this study were among a series of oxazolidinone derivatives synthesized and evaluated in our laboratory [8,9,10,11,12]. PH027 is a morpholine derivative similar to Lzd, while PH051 is a *N*-trifluoroacetylpiprazine derivative which probably caused the larger differences in the physicochemical properties and pharmacokinetic characteristics for this compound.

Lzd, PH027 and PH051 are relatively lipophilic compounds, with low water solubility. Different publications have reported different calculated Log *P* for Lzd, but they all range between 0.6–0.9, and the reported Lzd water solubility ranges between <1.0 mg/mL to 3 mg/mL [20,21,22,23]. We used molinspiration, which is a prediction software for calculation of the molecular properties to calculate the Log *P* for the three compounds [24]. Using this software, the calculated Log *P* for Lzd was 0.9, which is similar to the previously reported value for Lzd, and the calculated Log *P* values for PH027 and PH051 were 1.32 and 1.61, respectively. This indicates that PH027 and PH051 are more lipophilic than Lzd, and their water solubility is lower than that of Lzd. This was evident while preparing the 20% hydroalcoholic solution for IV administration, where Lzd was the fastest to dissolve, followed by PH027, and PH051 required slightly higher concentration of ethanol for complete dissolution. When the oral doses (20 mg/kg) of the three compounds were prepared, the three compounds did not dissolve completely in 20 mL of warm normal saline and the compounds were administered in the form of suspension, however it was clear that Lzd is more soluble than PH027, and PH051 is the least water-soluble of the three compounds.

The blood to plasma distribution ratios for Lzd and PH027 were similar and equal in unity, indicating that the binding of the two compounds in red blood cells and plasma are equal. Both compounds have approximately similar plasma protein binding; 32–34% for Lzd, and 37–38% for PH027, which is in the range of the reported 30% plasma protein binding for Lzd in humans [25]. While the blood to plasma distribution ratio for PH051 was lower than unity and its plasma protein binding is 91%. The low blood to plasma distribution ratio can result from higher plasma protein binding compared to the binding in the blood cells, and may also result from the presence of efflux transporter protein in the blood cells [19,26]. However, the high plasma protein binding of PH051 is the most probable cause of the unequal distribution between plasma and blood cells. High protein binding of drugs leads to low free drug concentration in plasma and reduces the drug in vivo activity since the free (unbound) drug is the active moiety of the drug [27]. So, the high plasma protein binding of PH051 significantly reduced its free concentration which can significantly contribute to its lower in vivo antibacterial activity. 

The three compounds showed a distribution phase followed by an elimination phase after a single IV bolus administration, however the distribution phase for Lzd and PH027 was very short, while that of PH051 was much longer. Lzd was eliminated slightly faster than PH027, while PH051 was eliminated at much slower rate. Among the three compounds under investigation, PH051 had the smallest AUC, the highest clearance, and the longest half-life. This suggests that PH051 has large volume of distribution and high tissue to plasma distribution ratios. The longer elimination half-lives for PH027 and PH051 compared to Lzd should lead to longer resident time of these compounds in the body, resulting in prolonged and usually higher activity. The fraction of Lzd dose excreted unchanged in urine after IV administration was only 5.7%, while that of PH027 is less than 0.5%, with only trace amounts of PH051 eliminated unchanged in urine. The fraction excreted unchanged in urine for these compounds decreased with the increase in lipophilicity, but the renal excretion of the three compounds in the rabbit were much lower than the reported 30% of Lzd dose excretion in urine in humans [25]. Metabolism is the main route of elimination of the three compounds under investigation in rabbits. Identification of the major metabolic products in urine samples obtained after the IV administration of the three compounds to rabbits showed that Lzd and PH027 are metabolized mainly through oxidation while PH051 undergoes amide hydrolysis (unpublished data from our laboratory). This may explain the difference in the elimination rate of the three compounds. The difference in the elimination pathways of the three compounds cannot explain the reduced in vivo activity of the novel compounds, since the reduced renal excretion should make higher fraction of the administered dose available for the systemic infection.

Lzd has been classified as a Class IV drug, based on the biopharmaceutical classification system (BCS), a class which includes drugs with low solubility and low permeability [28,29,30]. This is because Lzd has low water solubility and the reported Lzd Log *P* is lower than the 1.7 cut off value for highly permeable drugs [31,32]. The absorption of compounds that belong to Class IV after oral administration can improve by utilizing formulation strategies that improve the compound aqueous solubility and/or enhance its permeability across the gastrointestinal membrane [31,32]. Based on the BCS, PH027 and PH051 are also classifies as Class IV compounds since they have lower aqueous solubility than Lzd, and their calculated Log *P* is less than 1.7. Lzd is a weak base with reported p*K*a of 1.4. Co-administration of antacid does not have any effect on Lzd oral bioavailability in humans indicating that the ionization does not affect its absorption. Since the chemical structure of the novel compounds is not significantly different from that of Lzd, it is expected that they have p*K*a values in the same range of Lzd p*K*a. So, ionization should not play a significant role in the absorption of these novel compounds.

The oral bioavailability of Lzd in humans has been reported to be close to 100%, however the oral bioavailability of Lzd in the rabbit calculated from the IV and oral administration data was slightly less than 40% [25], while the oral bioavailability of PH027 and PH051 were 22%, and 4.7%, respectively. The incomplete bioavailability for the three compounds under investigation, most probably resulted from their poor water solubility, because their oral bioavailability improved when they were administered as microemulsion. The reason of the low oral bioavailability of Lzd in the rabbits compared to humans is not known, however, it is probably related to the limited solubility of Lzd oral dose in gastric contents. The total oral dose administered in our study to the rabbits was between 60–70 mg (20mg/kg), which is about 10% of the average human single oral dose (600 mg). So, it is possible that the solubility of Lzd in the rabbit GIT was lower because the rabbit GIT average volume is less than 10% of the human GIT volume [33,34].

The lower bioavailability of PH027 and PH051 compared with that of Lzd, is the most probable cause of the lower in vivo activity of the two novel compounds compared to Lzd, despite their comparable in vitro activity. In other words, although PH027 and PH051 have MIC values comparable to those of Lzd against Gram-positive resistant bacteria in vitro, the lower bioavailability of PH027 and PH051 resulted in lower amounts of these two compounds reaching the systemic circulation leading to decreased in vivo activity against systemic infection. As mentioned previously, the high plasma protein binding of PH051 and the resulting low free concentration is another contributing factor to its lower in vivo activity.

In an attempt to improve the bioavailability of the three compounds under investigation after oral administration, we administered the three compounds orally in the form of microemulsion. Microemulsions have been used before to improve the oral bioavailability of poorly soluble compounds [35]. We used SMEDDS, which consists of a mixture of surfactant, co-surfactant, and lipid, which has been used previously to improve the oral bioavailability of lipophilic drugs. The compounds were dissolved in SMEDDS made of Cremophor-EL, Solutol HS, Captex 355 (6:3:1). When the resulting solution is mixed with water microemulsion is formed [17]. The bioavailability of the three compounds increased when administered as microemulsion with the bioavailability of the more lipophilic and least soluble compounds PH027, and PH051 increasing by almost three-fold, while the bioavailability of Lzd increased by only 30%. The main objective of oral administration of the compounds in the form of microemulsion was to see if increasing the solubility of the three compounds by forming microemulsion, will improve their oral bioavailability. We used SMEDDS which have been used previously to prepare microemulsion for the three compounds under investigation, and we did not intend to optimize the formulation by choosing the best combination of surfactant, co-surfactant, and lipid for each compound. This may explain the difference in the improvement in oral bioavailability of the three compounds when administered as microemulsion. The improved bioavailability of the three compounds when administered as microemulsion, indicates that the poor bioavailability of these compounds when administered in the form of suspension in the rabbit most probably resulted from their poor water solubility. Improving the water solubility of these compounds will improve their systemic bioavailability, and possibly, the in vivo antibacterial activity.

The tissue to plasma distribution ratio should be determined when the drug in plasma and tissues is at equilibrium. Ideally, accurate determination of the tissue to plasma distribution is determined by comparing the concentrations in tissues and plasma at steady state. However, due to the poor aqueous solubility of three compounds under investigation, it was not possible to prepare aqueous solutions for IV infusion over 6–8 h to achieve steady state. So, the tissue to plasma distribution ratios were determined after IV bolus administration during the terminal elimination phase when the administered compound in all parts of the body is at equilibrium. Based on the plasma concentration-time profiles after IV administration of the three compounds, the tissue to plasma distribution was determined 2 h after the second IV bolus dose, when the distribution phase is completed. In general, the three compounds have good tissue penetration, which was evident from their tissue to plasma distribution ratios. Except for the brain, the tissue to plasma distribution ratios were approximately equal to one and higher, with the novel compounds having higher ratios in most investigated tissues probably because of their higher lipophilicity. The tissue to plasma distribution of PH051 was expected to be higher than that for PH027 and Lzd because of its high clearance and long half-life. So, it is possible that the tissue distribution of PH051 was underestimated because complete equilibrium between the plasma and tissues was not established. The higher tissue distribution of PH027 and PH051 compared to Lzd indicates that differences in the tissue distribution cannot be the cause of the reduced in vivo activity of the novel compounds compared to Lzd.

## 5. Conclusions

The novel oxazolidinone derivatives investigated in the current study, PH027 and PH051, have good in vitro activity against a variety of resistant bacteria, comparable to that of Lzd. The observed weak in vivo activity of these novel compounds against systemic infection after oral administration in the form of suspension most probably resulted from their poor oral bioavailability. The low oral bioavailability results in low systemic concentrations of the compounds and reduces their activity against systemic infections. It will be important to re-evaluate the in vivo activity of these novel compounds against systemic infection, using a formulation with improved oral bioavailability.

## Figures and Tables

**Table 1:** The blood to plasma partition and plasma protein binding for Lzd, PH027, and PH051 in rabbit blood.

Concentration	Linezolid	PH027	PH051
**Blood: plasma partition**			
250 μg/L *	0.973 ± 0.041 ^#^	0.96 ± 0.032 ^#^	0.772 ± 0.022 ^$^
1000 μg/L *	0.981 ± 0.011 ^#^	1.010 ± 0.021 ^#^	0.833 ± 0.015 ^$^
**Plasma protein binding (% bound)**			
250 μg/L *	34.2 ± 2.1	38.7 ± 3.1	91.1 ± 2.7
1000 μg/L *	32.2 ± 1.8	37.8 ± 2.4	90.9 ± 1.9

* *n* = 3, for each compound at each concentration; ^#^ Not significantly different from unity; ^$^ Significantly different from unity.

**Table 2:** Summary of the pharmacokinetic parameters for Lzd, PH027 and PH051 after IV and oral administration.

Parameters	Linezolid	PH027	PH051
**IV Administration (*n* = 4, 5 mg/kg)**			
AUC (μg h/L)	4038 ± 1280	5670 ± 933	1950 ± 986
CL_T_ (L/h/kg)	1.24 ± 0.43	0.882 ± 0.23	2.564 ± 1.212
Elimination rate constant (min^−1^)	0.0132	0.0101	0.00396
Half life (min)	52.4 ± 6.3	68.7 ± 12.1	175 ± 46.1
Renal clearance (L/h/kg)#	0.083 ± 0.016	0.00380 ± 0.0007	-
Fraction excreted unchanged in urine	5.70%	0.402%	-
**Oral Administration (*n* = 4, 20 mg/kg, as Suspension)**			
C_max_ (μg/L)	3910 ± 1010	1620 ± 820	69.7 ± 14.8
t_max_ (min)	60	60	60
AUC (μg h/L)	6250 ± 3620	5010 ± 2430	369 ± 378
Bioavailability (%)	38.7 %	22.1%.	4.73%
**Oral Administration (*n* = 3, 20 mg/kg, as Microemulsion)**			
C_max_ (μg/L)	3413 ± 1800	2871 ± 392	731 ± 412
t_max_ (min)	45	120	30
AUC (μg h/L)	8350 ± 3340	16,550 ± 3460	1083 ± 426
Bioavailability (%)	51.7%	72.9%	13.9%
Increase in bioavailability	33.6 %	328 %	293 %

^#^ Calculated from individual urine collection intervals, from renal excretion rate and plasma drug concentration.

**Table 3:** Tissue to plasma concentration ratio for Lzd, PH027 and PH051 after IV administration in rabbits.

Tissue	Tissue to Plasma Concentration Ratio *		
	Lzd	PH027	PH051
Kidney	2.05 ± 0.067	2.78 ± 0.089	2.22 ± 0.063
Brain	0.144 ± 0.015	0.223 ± 0.016	0.179 ± 0.07
Lung	1.24 ± 0.078	1.59 ± 0.041	1.503 ± 0.16
Liver	1.10 ± 0.013	2.91 ± 0.168	1.703 ± 0.19
Heart	0.857 ± 0.087	1.36 ± 0.087	1.561 ± 0.22
Muscles	1.21 ± 0.016	1.25 ± 0.059	1.581 ± 0.21
Spleen	0.725 ± 0.029	1.51 ± 0.026	1.41 ± 0.27
Eye (vetrus fluid) ^#^	0.886 ± 0.109	0.989 ± 0.10	0.810 ± 0.11

* ng/mL plasma:ng/gram tissue; ^#^ ng/mL plasma:ng/mL eye fluid.
